# Hybrid manifold smoothing and label propagation technique for Kannada handwritten character recognition

**DOI:** 10.3389/fnins.2024.1362567

**Published:** 2024-04-12

**Authors:** G. Ramesh, J. Shreyas, J. Manoj Balaji, Ganesh N. Sharma, H. L. Gururaj, N. N. Srinidhi, S. S. Askar, Mohamed Abouhawwash

**Affiliations:** ^1^Department of AIML-Artificial Intelligence & Machine Learning, Alva's Institute of Engineering and Technology, Mangalore, Karnataka, India; ^2^Department of Information Technology, Manipal Institute of Technology Bengaluru, Manipal Academy of Higher Education, Manipal, Karnataka, India; ^3^Department of Computer Science and Engineering, University Visvesvaraya College of Engineering, Bengaluru, Karnataka, India; ^4^Department of Computer Science and Engineering, Manipal Institute of Technology Bengaluru, Manipal Academy of Higher Education, Manipal, Karnataka, India; ^5^Department of Statistics and Operations Research, College of Science, King Saud University, Riyadh, Saudi Arabia; ^6^Department of Mathematics, Faculty of Science, Mansoura University, Mansoura, Egypt

**Keywords:** computer vision, convolutional neural networks, handwritten character recognition, machine learning, manifold smoothing, label propagation

## Abstract

Handwritten character recognition is one of the classical problems in the field of image classification. Supervised learning techniques using deep learning models are highly effective in their application to handwritten character recognition. However, they require a large dataset of labeled samples to achieve good accuracies. Recent supervised learning techniques for Kannada handwritten character recognition have state of the art accuracy and perform well over a large range of input variations. In this work, a framework is proposed for the Kannada language that incorporates techniques from semi-supervised learning. The framework uses features extracted from a convolutional neural network backbone and uses regularization to improve the trained features and label propagation to classify previously unseen characters. The episodic learning framework is used to validate the framework. Twenty-four classes are used for pre-training, 12 classes are used for testing and 11 classes are used for validation. Fine-tuning is tested using one example per unseen class and five examples per unseen class. Through experimentation the components of the network are implemented in Python using the Pytorch library. It is shown that the accuracy obtained 99.13% make this framework competitive with the currently available supervised learning counterparts, despite the large reduction in the number of labeled samples available for the novel classes.

## 1 Introduction

The challenge of converting manuscripts and printed documents into digital formats has been the focus of computer vision research (Nasir et al., 2021; Gowda and Kanchana, 2022). Recent advances have blurred the interface between physical copies of text and their digital counterparts. Large scale scanning of thousands of historical documents has been performed. Enabling visually impaired individuals to read signboards and paper, and faster processing of checks. Legislative bodies have benefited from the ease of digitizing legal documents, allowing for seamless transfer, signing, and searching. The field of handwritten character analysis has strive to make effective algorithms to achieve various goals, such as the classification of handwritten characters, the classification of the writers of different manuscripts, generating text matching the handwriting of a writer, and so on (Dhiaf et al., 2023). Prior to supervised deep neural networks, handcrafted methods were used for handwritten character recognition, which often required several different steps such as binarization of images, rescaling and rotating the images, performing statistical aggregations on different parts of the images, etc. This required fine-tuning a large number of parameters to obtain accurate results and could not generalize well to variations in the input images (Aradhya et al., 2010; Ramesh et al., 2020).

Supervised learning using deep neural networks has allowed most of the explicit tasks to be replaced by a single neural network model that, by virtue of back-propagation, is able to learn the weights required for effective extraction of features from the images that are used for classification. By providing a large training set that includes diverse samples of each character, the neural network is rendered more robust in its accuracy in classifying a larger range of handwriting samples. However, the creation of a large labeled training set of images is laborious, and certain character classes have few real-world samples. By utilizing already pre-trained models to predict the new classes, sample efficiency is improved. The difficulty in obtaining such a dataset for Kannada handwritten characters is compounded by the large number of possible graphemes in the Kannada script, stemming from the use of combinations of base characters to form digraphs. Semi-supervised learning techniques, which exploit the use of a large unlabeled dataset to improve the robustness and accuracy of a model trained on a small labeled training set, have been successfully used to achieve this goal. The scenario of novel classes' incorporation is modeled with the episodic learning approach (Nichol et al., 2018). Recent works in few shot learning make use of this framework to mimic meta-learning tasks (Gidaris et al., 2019). Improved generalization of the neural network is achieved through the use of data augmentation where sample images are rotated in four different orientations, increasing the number of training samples the network is trained on (Zhou et al., 2004). The use of label propagation allows the incorporation of new classes into the classification framework with very few extra training samples (Alsuhibany and Alnoshan, 2021). The handwritten CAPTCHA image then asks visitors to choose the joints between Arabic letters. In the latter approach, a novel generator of Arabic handwritten CAPTCHA pictures is devised; once the image is formed, the user is required to input the letters depicted in the image (Weldegebriel et al., 2019). Although both have showed encouraging outcomes, this experimental study compares both in terms of security and usability for mobile device applications.

The enormous success of supervised neural network-based machine learning approaches can be ascribed to the minimal amount of manual parameter adjustment needed as well as the models' flexibility to learn efficient feature representations that work for a variety of inputs. However, supervised neural network models need well-curated, sizable, labeled datasets to obtain strong generalization capabilities and robustness. This makes it feasible for the models to accurately learn the various potential variations they might experience. Due to the bias introduced by unbalanced datasets, these models may favor predicting the classes that were represented more frequently in the training set, which would lead to subpar performance when identifying previously undiscovered classes of characters. Being one of the acknowledged regional languages in India, Kannada also serves as the province of Karnataka's official language of communication (Ramesh et al., 2019b). The literature and artistic diversity of the language makes it a priceless repository of information and culture. Many of these regional languages need the power of technology to retain the language directed at them (Thippeswamy and Chandrakala, 2020; Parikshith et al., 2021). The preservation of the language's scripture is greatly aided by advances in digitization, which also give the language a significant edge in terms of reaching a wider audience given the pervasiveness of internet access around the world. Building precise pattern recognition models is also a difficult task due to the absence of readily accessible annotated data relevant to the local languages. The suggested study addresses the issue of “Recognizing Kannada Handwritten Characters in a Few-Shot Learning viewpoint” by utilizing a strong, cutting-edge technique that offers best-in-class accuracy and consistent outcomes. There are 47 basic characters in the Kannada alphabet. Main contribution of this paper are as fallows, we introduce a Manifold Smoothing and Label Propagation-based Approach for Offline Handwritten Kannada Character Recognition. In particular, our contributions are outlined as follows: The goal of this work is to combine a few techniques in order to create an offline Kannada handwritten character classifier that can be trained to retain high accuracies on classes with as few as one or five samples. This allows for the rapid incorporation of classes with minimal extra samples required.

- A novel classes incorporation is modeled with the episodic learning approach.
- Improved generalization of the neural network is achieved through the use of data augmentation.
- The label propagation allows the incorporation of new classes into the classification framework with very few extra training samples.

## 2 Related work

Weldegebriel et al. (2019) presented by the Handwritten Ethiopian Character Recognition (HECR) dataset was used to prepare a model, and the HECR dataset for images with more than one shading pen RGB was considered. This framework employs a half breed model comprised of two super classifiers: CNN and eXtreme Gradient Boosting (XGBoost). CNN-XGBoost characterization error rate brings about HECR dataset 0.1612%. This proposed work got an accuracy of 99.84% in the CNN-XGBoost strategy. Sahlol et al. (2020) proposed a hybrid ML approach that uses area binary whale improvement calculation to choose the most suitable highlights for the recognition of handwritten Arabic characters. This strategy utilized the CENPARMI dataset and This strategy results show away from of the proposed approach as far as memory footprint, recognition accuracy, and processor time than those without the features of the proposed technique. This proposed BWOA-NRS approach beats any remaining works in both execution and time utilization got an accuracy of 96% in 1.91 s time. Cilia et al. (2018) has considered various univariate measures to create an feature ranking and proposed a greedy search approach for picking the element subset ready to maximize the characterization results. One of the best and broadly set of features in handwriting recognition and we have utilized these features for considering to tests of three genuine word information bases. Karthik and Srikanta Murthy (2018) presented by the recognition of isolated handwritten characters of Kannada proposed a new method based on deep belief network with DAG features. The recognition accuracy for consonants and vowels to achieve an accuracy of 97.04% using deep belief network.

Weng and Xia (2019) proposed technique using Convolutional neural network has been approved by previous work with the results of existing strategies, utilized for optical character recognition. In this strategy, First, build a Shui character dataset for applying a Convolutional neural network to manually written character recognition, at that point during the proposed of the CNN, analyzed the consequences of various parameters so that proposed the parameter tuning suggestions and accuracy is around 93.3%. Guha et al. (2019) presented by CNN has been a well-known way to deal with remove features from the image data. in this work, we consider as different cnn models freely accessible Devanagari characters and numerals datasets. This method uses a Kaggle Devanagari character dataset, UCI character dataset, CVPR ISI Devanagari dataset, and CMATERdb 3.2.1 dataset. Using the DevNet, the recognition accuracies obtained on UCI DCD, CVPR ISI Devanagari character dataset, CMATERdb 3.2.1, and Kaggle Devanagari character dataset have obtained an accuracy of 99.54, 99.63, 98.70, and 97.29%, respectively. Khan et al. (2019) proposed technique presents a efficient handwriting identification framework which joins Scale Invariant Feature Transform (SIFT) and RootSIFT descriptors in a bunch of Gaussian mixture models (GMM). This proposed system using six different public datasets are IAM dataset obtained accuracy of 97.85%, IFN/ENIT dataset obtained an accuracy of 97.28%, AHTID/MW dataset obtained an accuracy of 95.60%, CVL dataset obtained an accuracy of 99.03%, Firemaker dataset obtained an accuracy of 97.98%, and ICDAR2011 dataset obtained an accuracy of 100.0%.

Sahare and Dhok (2018) proposed robust algorithms for character segmentation and recognition are introduced for multilingual Indian document images of Latin and Devanagari contents. Perceiving the input character utilizing the KNN classifier technique, as it has characteristically zero preparing time. This strategy got the highest segmentation and recognition rates of 98.86% is acquired on an exclusive information base of Latin content and the Proposed recognition algorithm shows the most best accuracy of 99.84% on the Chars74k numerals data set. Zheng et al. (2019) proposed strategy separate a novel component from pooling layers, called uprooting highlights, and join them with the features coming about because of max-pooling to catch the structural deformations for text recognition tasks. This strategy utilizes three content datasets, MNIST, HASY, and Chars74K-textual style, and contrasted the proposed technique and CNN based models and best in class models. Mhiri et al. (2018) work depends on deep CNN and it doesn't need explicit segmentation of characters for the recognition of manually written words. Proposed strategy presentation forward and in reverse ways or robust representation. This proposed approach use IAM and RIEMS information base and this methodology achieve a word error rate of 8.83% on the IAM information base and 6.22% on the RIEMS dataset.

Sueiras et al. (2018) proposed a technique framework for recognizing offline handwritten words and use of another neural architecture design that consolidates a deep cnn with an encoder-decoder, called sequence to sequence. This proposed technique utilizes two handwritten databases are IAM and RIMES datasets and these datasets acquire a word error rate in the test set of 12.7% in IAM and 6.6% in RIMES datasets. Katiyar and Mehfuz (2016) proposed presents hybrid feature extraction and GA based feature selection for off-line handwritten character recognition by utilizing adaptive MLPNN classifier. The proposed technique has been performed utilizing the standard database of Center of Excellence for Document Analysis and Recognition for the English alphabet. It is obvious from the outcomes that the proposed strategy beats the other state of art techniques with an accuracy of 91.56 and 87.49% individually for capital alphabet and little alphabet in order (Singh et al., 2020). The proposed technique contains six non-Indic-contents and eight Indic contents specifically, Persian, Roman, Thai, Chinese, Japanese, Arabic, Chinese, Japanese Assamese, Bangla, Devanagari, Gurmukhi, Tamil, Telugu, Kannada, and Malayalam. This strategy conversation about the classification tools, pre-processing steps, include feature extraction, and approach's utilized, and different online handwriting recognition methods advancement have been carried out. Ramesh et al. (2019a) demonstrate the use of Convolutional Networks in generating extremely accurate handwritten character classifiers. They assembled the vowels and consonants freely and utilized 400 images for each character for preparing the CNN. They have claimed the accuracy of 98.7%.

## 3 System architecture

**Figure 1:**
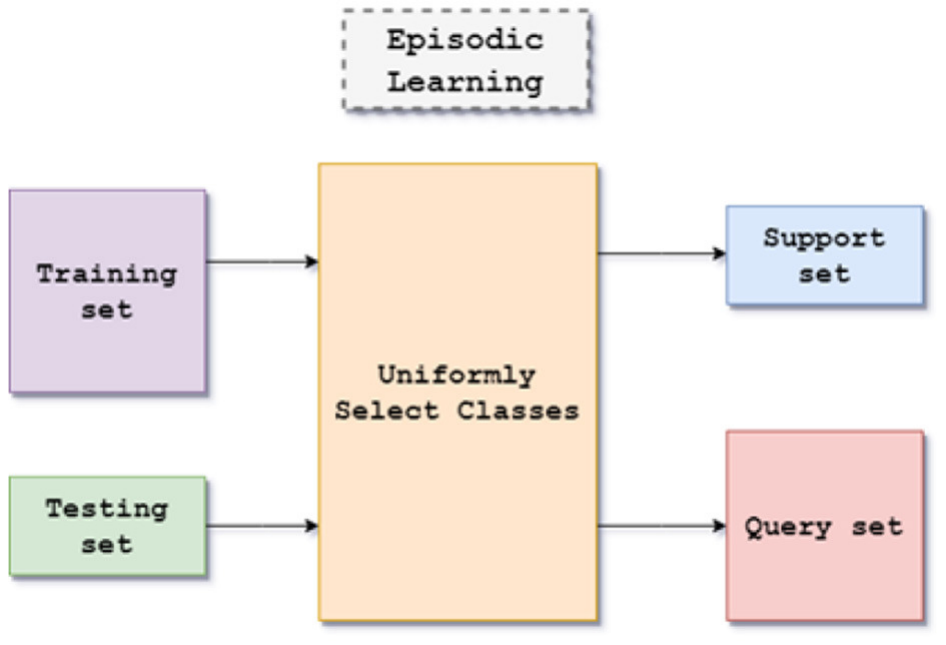
Flow diagram of episodic framework.

The proposed method's architecture is based on the episodic framework for few-shot learning shown in Figure 1. The dataset consists of images of handwritten characters in Kannada with 400 examples, written by multiple writers each for 47 classes with a size of 84 x 84 for each image. The episodic framework is utilized to evaluate the architecture in a few-shot environment.

### 3.1 Experimental steps

The experiment is carried out with the following steps:

- **Collection of the dataset:** The dataset consists of 47 classes representing each base character of the Kannada abugida. Each class consists of 400 samples obtained from different writers. 50% of the dataset is used for pretraining (24 classes), 2% is used for finetuning (12 classes), and 25% is used for the validation set (11 classes).
- **Preprocessing the images:** The images are rescaled to 84 x 84px using the Python Image Library (PIL) library. Bilinear interpolation is used to achieve this. The images are converted to RGB format.
- **Training the handwritten character classifier:** Two different convolutional networks are used, the Conv4 network and the Resnet-12 network. The training consists of the pretraining phase where the network is trained on the base set. The next phase is the finetuning phase, where the network is trained in an episodic fashion on the unseen classes.
- **Analyzing the result:** The accuracy and loss of the two different networks are plotted and compared. Training and Validation accuracy are plotted for the pretraining phase (seen characters), while Test and Validation accuracy are plotted for the finetuning phase. 1-shot and 5-shot finetuning are performed (one example per class and five examples per class, respectively).

### 3.2 Episodic framework

The episodic framework was introduced by Nasir et al. (2021). It provides a simulation for training a meta-learning model for few-shot classification tasks. In the episodic framework is a large labeled dataset C_train_ is present. The goal is to train the classifier on a previously unexplored set of classes C_test_, where there are only a few labeled samples available. To create a support set S and query set Q for each episode, a small subset of N classes from the C_train_, each task has N classes that need to be classified in N way K shot learning, which has K available labeled samples. In contrast to the query set Q's different examples from the same N classes, the support set S's K examples from each of the N classes. In this work, N = 5 classes are chosen, and the size of the query set is 15 examples per class. The five classes are chosen uniformly over the union of sets (C_train_) U (C_test_) and sample accordingly. A transductive setting is used due to the small size of K in the support set. The entire query set Q can be used for predicting labels rather than predicting each example independently. This helps alleviate the bias caused by the small number of samples while improving generalization.

## 4 Proposed approach

The proposed work uses the combination of manifold smoothing and label propagation to solve the considered problem statement. For better generalization, Manifold Smoothing is used to regularize the features extracted for better generalization, while Label Propagation allows few-shot inference on unseen classes.

### 4.1 Manifold smoothing with metric learning

In order to make the decision boundaries of the hidden layer of the model more smooth, resulting in better robustness and generalization, a layer to smoothen the extracted features is used (Lee et al., 1995). Given the feature vectors z_i_ ∈ *R*^*m*^ (*R*^*m*^ is the set of m-dimensional real number vectors) which are extracted using the Convolutional Neural Network layers, a smoothing function is applied to obtain the smoothed feature vectors ${\tilde{z}}_{\text{i}}$, which are forwarded to the fully connected layer for classification. This smoothing process consists of using a Gaussian similarity function using the L2 norm as a measure of the similarity/dissimilarity of the different features. $d_{\text{ij}}^{2}=∥$z_i_-z_j_${∥}_{2}^{2}$ where $d_{\text{ij}}^{2}$ is the distance between feature vectors z_i_ and z_j_ and ∥z_i_-z_j_${∥}_{2}^{2}$ is the square of the L2 norm between the feature vectors, for pairs of features z_i_, z_j_ and

A similarity matrix is constructed using Equation 1:


(1) $$\begin{array}{l} A_{\text{ij}}=e^{\frac{-d_{\text{ij}}^{2}}{\sigma^{2}}} \end{array}$$

where A_ij_ is the element of the similarity matrix A, $d_{\text{ij}}^{2}$ is the distance between feature vectors z_i_ and z_j_ and $\sigma^{2}=\text{Var}(d_{\text{ij}}^{2}$) is the variance of $d_{\text{ij}}^{2}$.

The similarity matrix A is normalized using the Laplacian in order to ensure convergence:


(2) $$\begin{array}{l} L=D^{-\frac{1}{2}}AD^{-\frac{1}{2}}, \end{array}$$

where L is the Laplacian similarity matrix computed using normalizing matrix D defined as Equation 3.


(3) $$\begin{array}{l} D_{\text{ii}}=\underset{\text{j}}{\sum }A_{ij} \end{array}$$

Power iteration is used to successively increase the weights of the closest features while reducing the weights of the features that are not too close to each other. This is similar to the power iteration needed in label propagation, and the propagator matrix P is thus obtained by:


(4) $$\begin{array}{l} P={\left(I-\alpha L\right)}^{-1} \end{array}$$

where P is the propagator matrix, I is the identity matrix, α is the smoothing factor and L is the Laplacian obtained using Equation (2). The new feature vectors are calculated as Equation 5:


(5) $$\begin{array}{l} {\tilde{z}}_{\text{i}}=\underset{\text{j}}{\sum }P_{\text{ij}}z_{j} \end{array}$$

where P is the matrix calculated in Equation (4), z_j_ is the input feature vector and ${\tilde{z}}_{\text{i}}$ is the smoothed feature vector.

This is similar to a weighted sum of neighbors, resulting in a reduction in the noise present in each feature vector.

### 4.2 Label propagation

The prediction of labels for the query set Q using label propagation is obtained using the similarity matrix that is equivalent to the one used in the manifold smoothing step. Given the query set Q, the equation for the label matrix Y is given by:


(6) $$\begin{array}{l} Y=\frac{Y_{S}}{0} \end{array}$$

where Y is the label matrix,

- The matrix Y_S_ of size (nk × n) corresponds to the support set S. In each row of Y_S_, the column corresponding to the correct label is 1, (Y_ij_ = 1) if y_i_ = j. The rest of the elements are 0.
- The matrix 0 is a matrix of 0s of size (t × n) and corresponds to the query set Q. n is the number of classes, k is the number of samples per class in S, and t is the number of samples in Q.

Label propagation iteratively determines the unknown labels for the union set S ∪ Q (Ramesh et al., 2020):


(7) $$\begin{array}{l} F_{\text{t}+1}=\alpha LF_{\text{t}}+\left(1-\alpha \right)Y \end{array}$$

where L is the normalized similarity matrix calculated in Equation (2), F_t_ is the label propagation after t iterations, Y is the label matrix defined in Equation (6) and α is the smoothing factor between 0 and 1. The sequence F_t_ converges to


(8) $$\begin{array}{l} F^{*}={\left(I-\alpha L\right)}^{-1}Y \end{array}$$

where F^*^ is the matrix obtained on convergence of Equation (7) as t → ∞. The different features are clustered in a similar fashion to graph spectral clustering Equation (8).

### 4.3 Feature extraction using convolutional neural networks

The features are extracted from the input images using convolutional neural network layers (CNNs). Two CNN feature extractors are used in the experiments to determine the one with greater efficacy.

- The first feature extractor is a standard CNN Model with four layers Each layer consists of a convolution (kernel of size 3 × 3), as mentioned in Table 1 followed by Max-Pooling which reduces the size of the image progressively in each layer. The window of the Max-Pool layer is (2 × 2). The ReLU (Rectified Linear Unit) is used as the activation function which zeroes negative values.

**Table 1:** Layer of Conv4 network.

**Layer name**	**Output shape**	**Next layer**
Input layer	(84, 84, 3)	Conv0
Conv0	(42, 42, 64)	Conv2
Conv2	(10, 10, 64)	Conv3
Conv3	(5, 5, 64)	AvgPool
AvgPool	(64)	Output

The second is a Resnet Model with 12 layers (Karthik and Srikanta Murthy, 2018). This model is deeper, and each block has an identity shortcut path that helps prevent the vanishing gradient problem that is exacerbated as the number of layers increases. This increased depth improves the feature representation of the model, resulting in greater accuracy.

As mentioned in Table 2 each block has 3 convolutional layers, a shortcut connection between the first and the third layer and a Max-Pool layer (of window (3 × 3)). The shortcut connection adds the output of the first layer and third layer before passing it to the activation function (ReLU again).

**Table 2:** Layer of RestNet12 network.

**Layer name**	**Output shape**	**Next layer**
Input layer	(84, 84, 3)	Block0
Block0	(26, 26, 64)	Block1
Block1	(9, 9, 128)	Block2
Block2	(3, 3, 256)	Block3
Block3	(512)	Output

### 4.4 Pretraining process

**Figure 2:**
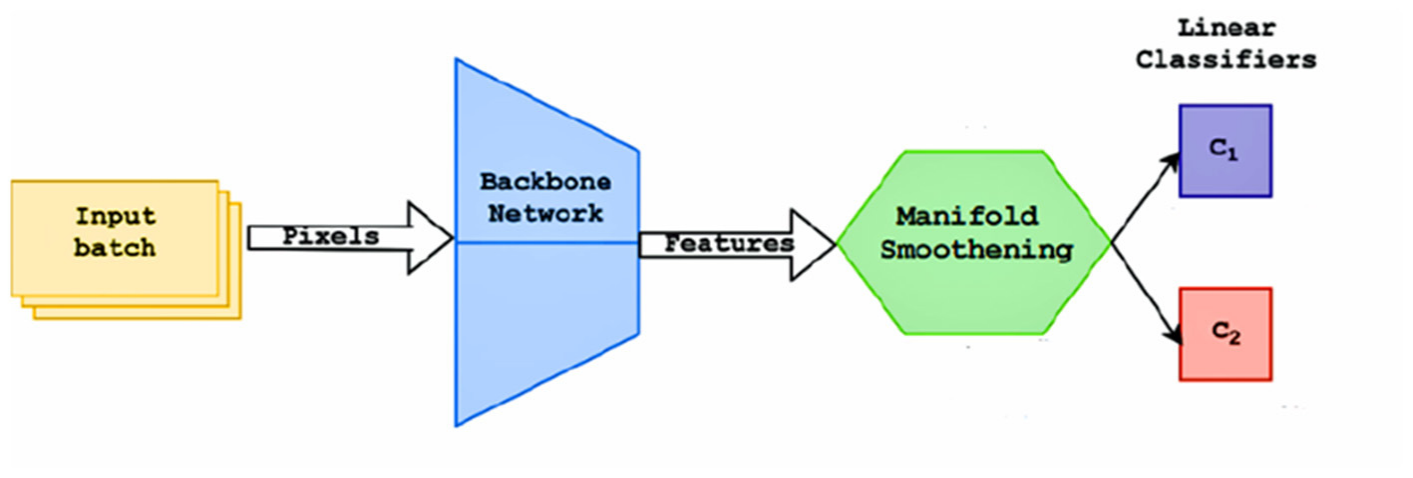
Flow diagram of the pretraining process.

The pretraining process is similar to a supervised training schedule. The training set C_train_, It contains classes that have a large number of labeled examples. The objective of the pretraining phase is to learn a good feature representation of the images, which can later be fine-tuned to classify unseen classes. Input batches of size 128 are used to improve the efficiency of batch normalization (He et al., 2016), reducing overfitting and improving the smoothness of gradients. Each image is rotated four times for the self-supervision loss (Dhiaf et al., 2023). Stochastic Gradient Descent is used to train the network. The pretraining process is defined in Algorithm 1. Two fully connected classifiers are trained as shown in Figure 2, which use the features extracted by the CNN backbone networks and regularized using the manifold smoothing process.

- The first classifier C1 is trained to predict the class labels of the input images. A standard cross entropy loss for classification is used to train this classifier.

The loss function is given by Equation (9):

(9) $$\begin{array}{l} L_{\text{C}1}\left(x_{i},y_{i};W_{l},\theta \right)=-lnp\left(\left(y_{i}|{\tilde{z}}_{\text{i}}\right),W_{l}\right) \end{array}$$

- The second classifier C2 is utilized to provide a self-supervision type learning signal, where the rotation angle of each input image (after being rotated by 0°, 90°, 180°, 270°), is predicted. This helps improve the learning signal and provides a certain degree of rotation invariance to the model.

The loss function is given by:

(10) $$L_{C2}(x_{i},y_{i};W_{\gamma },\theta )=-lnp(r_{i}|{\tilde{z}}_{t}),W_{\gamma })$$

where W_γ_ is the fully connected layer with softmax activation representing C_r_ and r_i_ is the prediction of the rotation angle.
The overall loss to be minimized is given by:

(11) $$\begin{array}{l} \text{argmin}\overset{128}{\underset{i=1}{\sum }}\overset{4}{\underset{j=1}{\sum }}L_{\text{C}1}\left(x_{i},y_{i};W_{l},\theta \right)+L_{\text{C}2}\left(x_{i},y_{i};W_{\gamma },\theta \right) \end{array}$$

where L_C1_ (x_i_,y_i_;W_l_,θ) is defined in Equation (10), L_C2_ (x_i_,y_i_;W_l_,θ) is defined in Equation (11) and argmin optimizes the arguments to minimize the sum.

**Algorithm 1:**
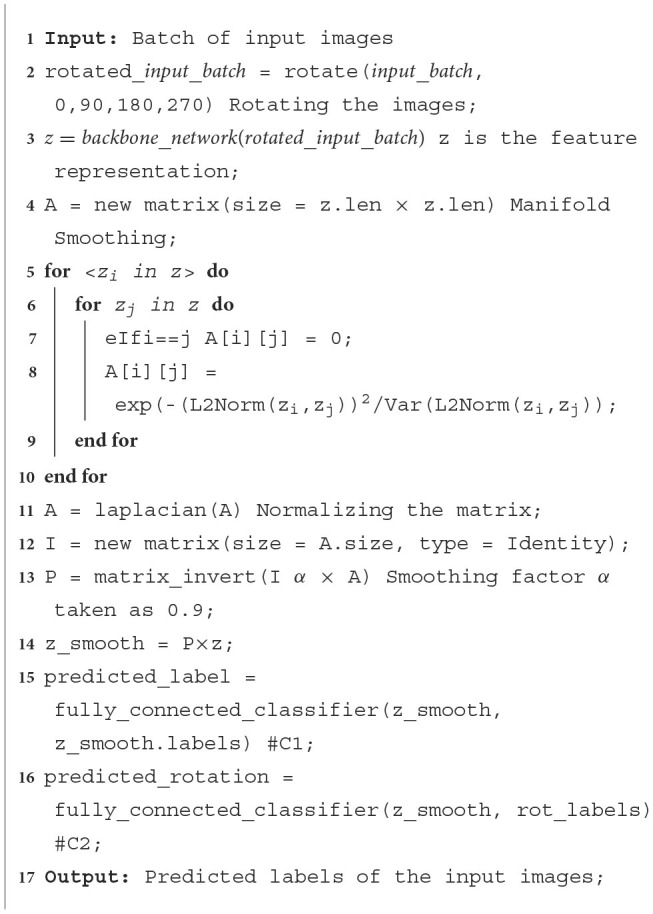
Pretraining algorithm.

### 4.5 Finetuning process

**Figure 3:**
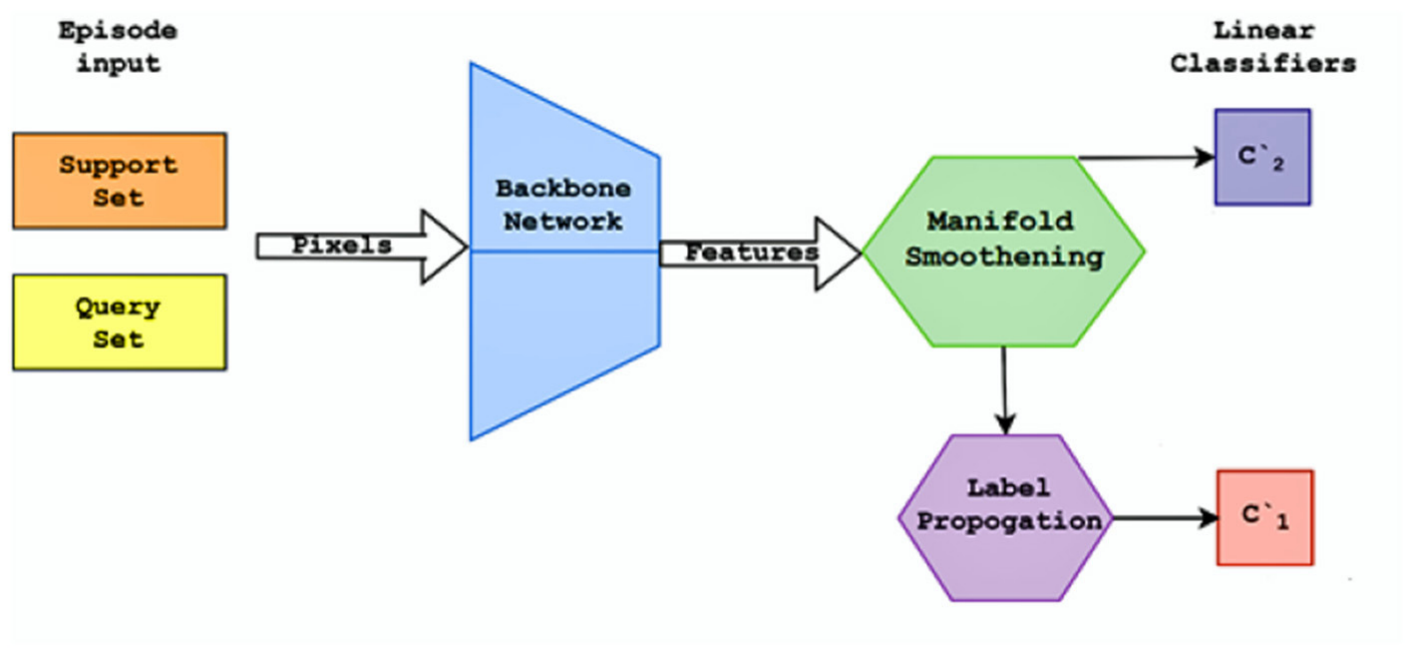
Flow diagram of the finetuning process.

The finetuning process is performed after the model has been trained on the training set C_train_. Here, the objective is learning to recognize the unseen classes (part of the test set C_test_. The label propagation method is used to find the labels of the unseen classes. Each epoch in finetuning consists of generating an episode calculating the loss obtained and using backpropagation to adjust the weights accordingly. The finetuning process is defined in Algorithm 2, Two linear classifiers are once again used as shown in Figure 3.

1. The classifier C‘_1_ utilizes label propagation to compute the probabilities of the classes in the query set. The logits are converted to class probabilities using the SoftMax function.

The loss function is given by Equation (12):

(12) $$\begin{array}{l} L_{\text{C‘}1}\left(x_{\text{i}},y_{\text{i}};\theta \right)=-lnp\left(y_{\text{i}}|\left(\tilde{z_{\text{l}}}\right),\tilde{Z},Y_{\text{S}}\right) \end{array}$$

where x_i_ is the input image, y_i_ is the label of the input image, θ is the CNN feature extractor and -ln p(y_i_∣ z_i_,$\tilde{Z}$, Y_S_) is the cross-entropy loss defined on predictions using label propagation (Y_S_) defined in Section V.

2. Since the label propagation loss tends to favor mixing of features, impacting the discriminativeness of the feature representation, a second classifier C‘_2_ is trained with the standard cross entropy loss on the union S∪Q. This helps in preserving the discriminativeness of the feature representation.

**Algorithm 2:**
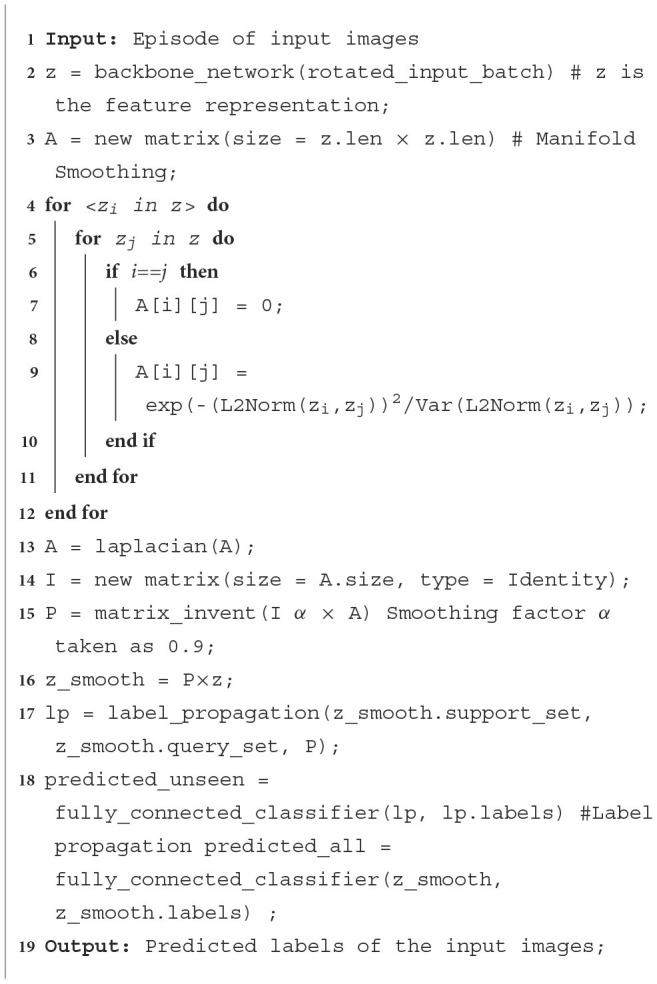
Finetuning algorithm.

The loss function is given by


(13) $$\begin{array}{l} L_{\text{C‘}2}\left(x_{\text{i}},y_{\text{i}};W_{\text{l}},\theta \right)=-lnp\left(y_{\text{i}}|\tilde{z_{\text{l}}},W_{\text{l}}\right) \end{array}$$

The overall loss to be minimized is the additive combination of the above:


(14) $$\begin{array}{r} argmin[\frac{1}{\left|Q\right|}\sum_{(x_{i},y_{i})\epsilon Q}L_{C\text{'}1}(x_{i},y_{i},\theta )+\frac{1}{\left|S\cup Q\right|}\cup \sum_{(x_{i},y_{i})\epsilon S\cup Q}\;\\ \cap \frac{1}{2}L_{C\text{'}2}(x_{i},y_{i};\cap W_{l},\theta )] \end{array}$$

Where Q is the query set, S is the support set, L_C‘1_(x_i_,y_i_,θ) is defined by Equation (13), L_C‘2_(x_i_,y_i_; W_l_,θ) is defined by Equation (14) and argmin optimizes the arguments to minimize the given sum.

## 5 Implementation

This work uses the dataset used in Karthik and Srikanta Murthy (2018) to evaluate the model. The components of the network are implemented in Python using the Pytorch library. The Episode Generator is used to create episodic tasks for the finetuning of the network. The backbone networks are assigned to the GPUs using the CUDA directive. The model's hyperparameters are listed.

### 5.1 Simulation dataset

The dataset consists of 47 classes representing each base character of the Kannada abugida. Each class consists of 400 samples obtained from different writers. The images are rescaled to 84 × 84 px using the PIL library. For the purpose of the experiments, the 47 classes are randomly split into three sets following the example of He et al. (2016). The base set C_train_ consists of 24 classes and has all 400 samples for the supervised pretraining phase. Thus 50% of the dataset is used for the supervised training part. A mixture of vowels and consonants are present in C_train_. Characters with shapes both simple and complex are represented in the training set.

The novel set C_test_ consists of 12 classes which form the unseen set of classes used to test the finetuning approach. This is 25% of the dataset. It is observed that characters both similar in shape to the ones found in C_train_, as well as uniquely shaped characters can be found in C_test_. A validation set C_val_ consisting of 11 classes is used to form the validation set used for hyperparameter search and to measure the amount of overfitting. Twenty-five percent of the dataset is used for this purpose.

## 6 Results and analysis

State of the art results is achieved using the Label Propagation and Manifold Smoothing model for the problem of Recognition of Handwritten Kannada Characters in a Few-Shot Learning perspective. This section gives insights of the result obtained in terms of Pretraining Accuracy (seen classes), Finetuning accuracy (seen and unseen classes) using 1-shot and 5-shot learning (support set of one and five examples, respectively). Comparison of result with the existing work is done here.

### 6.1 Performance evaluation

Two different feature extractors are evaluated using the episodic framework, and the average accuracy of classification over 1,000 episodes is used as the metric for evaluation. The first feature extractor, Conv4, has a faster training and inference time owing to its simplicity, and seems to benefit much more from the finetuning phase as compared to the second feature extractor, Resnet-12. However, much better accuracy is obtained by the larger Resnet-12 network. This can be attributed to the greater width of the network, which allows a larger number of learnable parameters to be used for classification. Although there is a greater amount of overfitting as evidenced by the difference in test and validation accuracies, the performance on finetuning shows that the framework has good generalization capability.

### 6.2 Conv4 network

The convergence of training at 44 epochs is observed, and due to the episodic nature of training, large swings are seen prior to convergence. The loss is monotonically decreasing over a large number of epochs, with a bump close to the convergence point.

**Figure 4:**
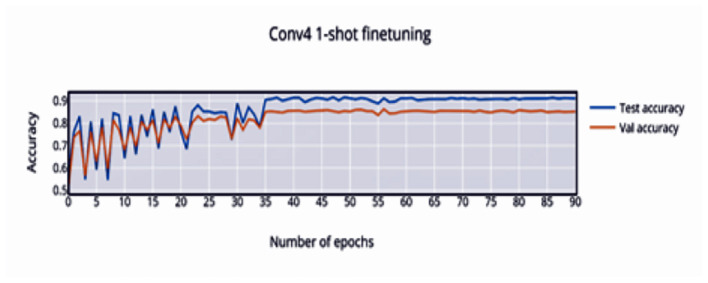
1-shot finetuning accuracy vs. number of epochs.

**Figure 5:**
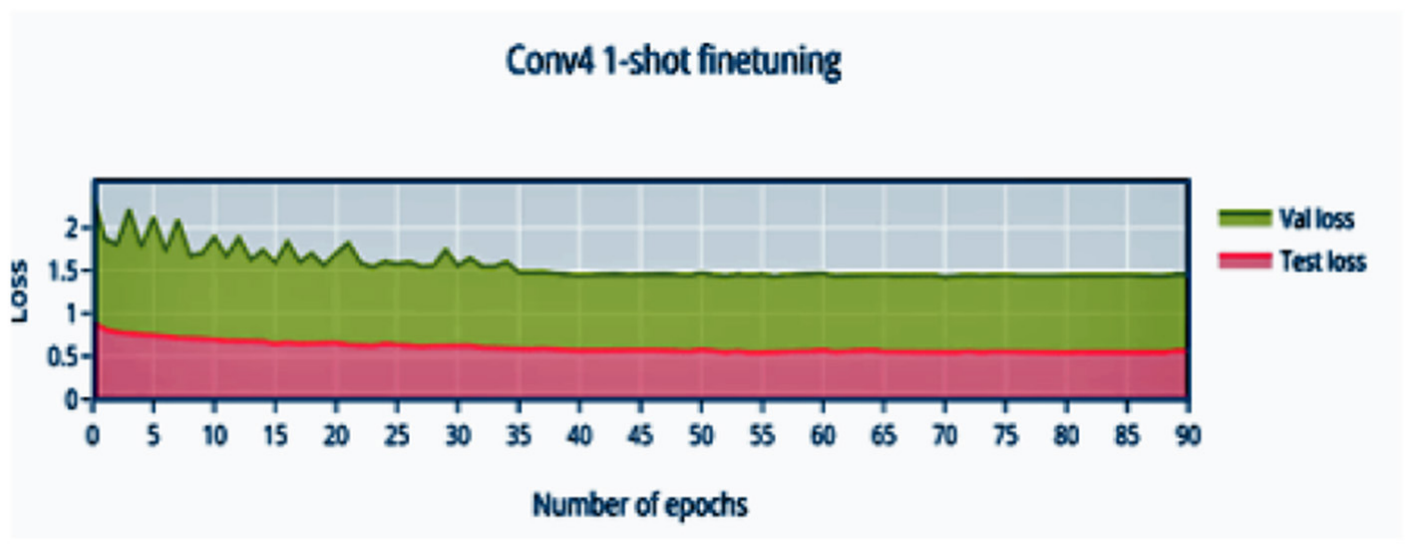
1-shot finetuning loss vs. number of epochs.

In Figure 4 it is observed that the pretrained model starts out at 50% accuracy and steadily increases with finetuning epochs until epoch 32 where the network converges to 91.04% accuracy. The loss (Figure 5) decreases and stabilizes.

**Figure 6:**
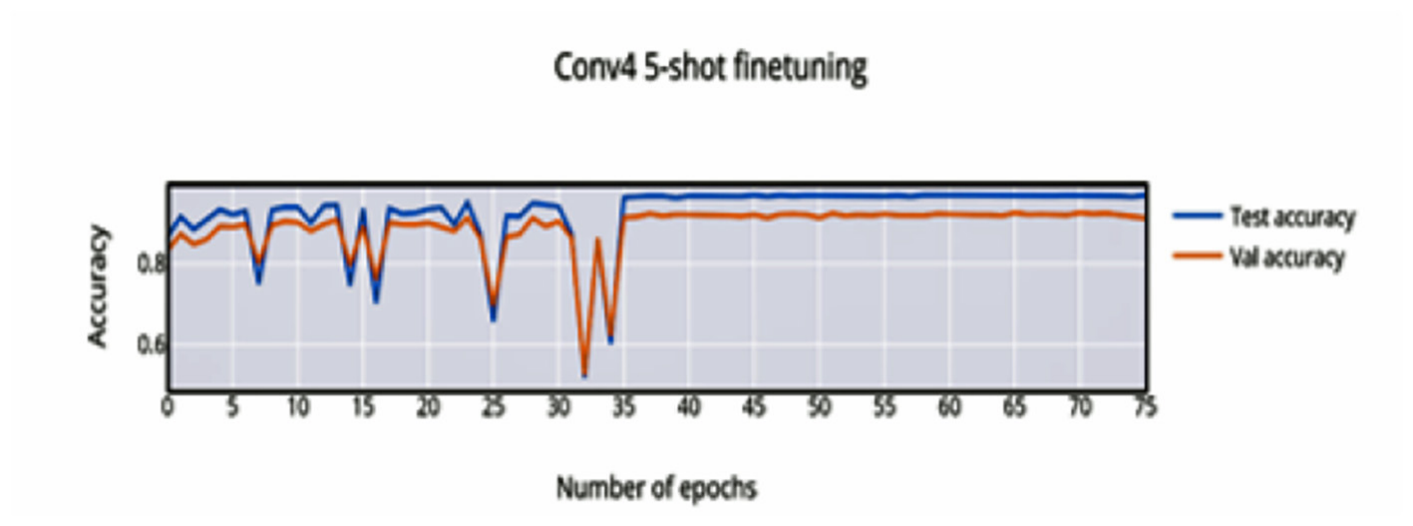
5-shot finetuning accuracy vs. number of epochs.

**Figure 7:**
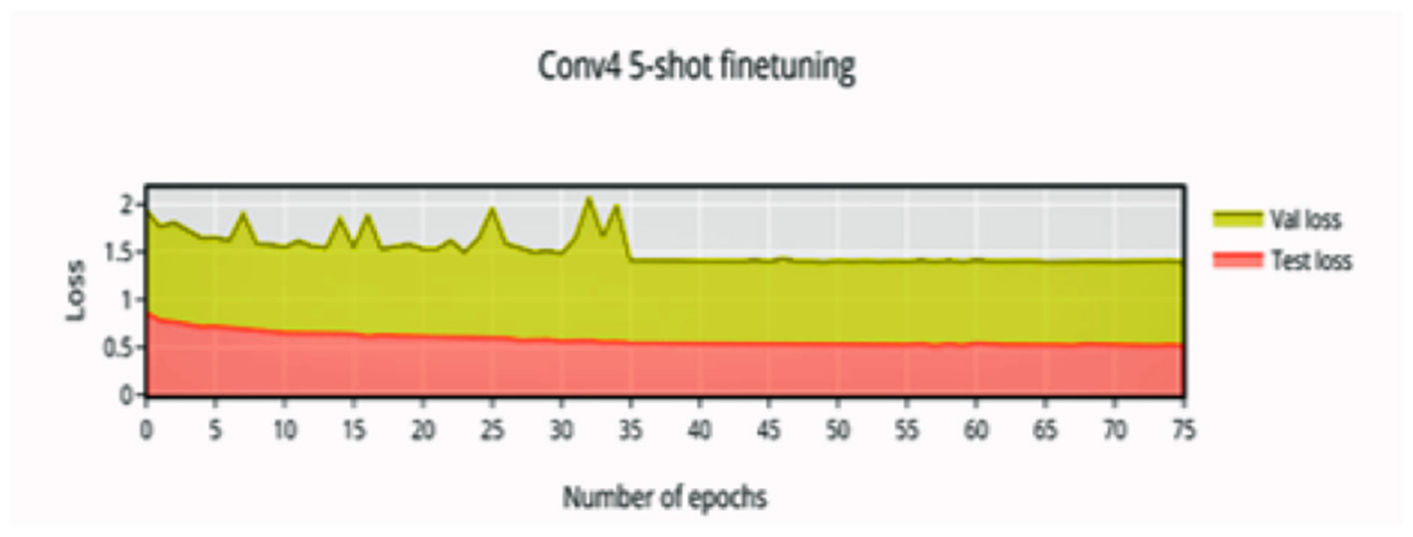
5-shot finetuning loss vs. number of epochs.

In 5-shot finetuning, a higher initial accuracy of 83% accuracy (Figure 6) is observed which reduces when more unseen classes are initially encountered, the network finally converges at 37 epochs to an accuracy of 96.88%. There is an increase in validation loss (Figure 7) corresponding to the more difficult episodes.

### 6.3 ResNet-12 network

The shorter convergence time (35 epochs) is seen and a higher pretraining accuracy being achieved (98.66%). This can be attributed to the increased number of channels (width) and layers (depth) of the backbone network.

**Figure 8:**
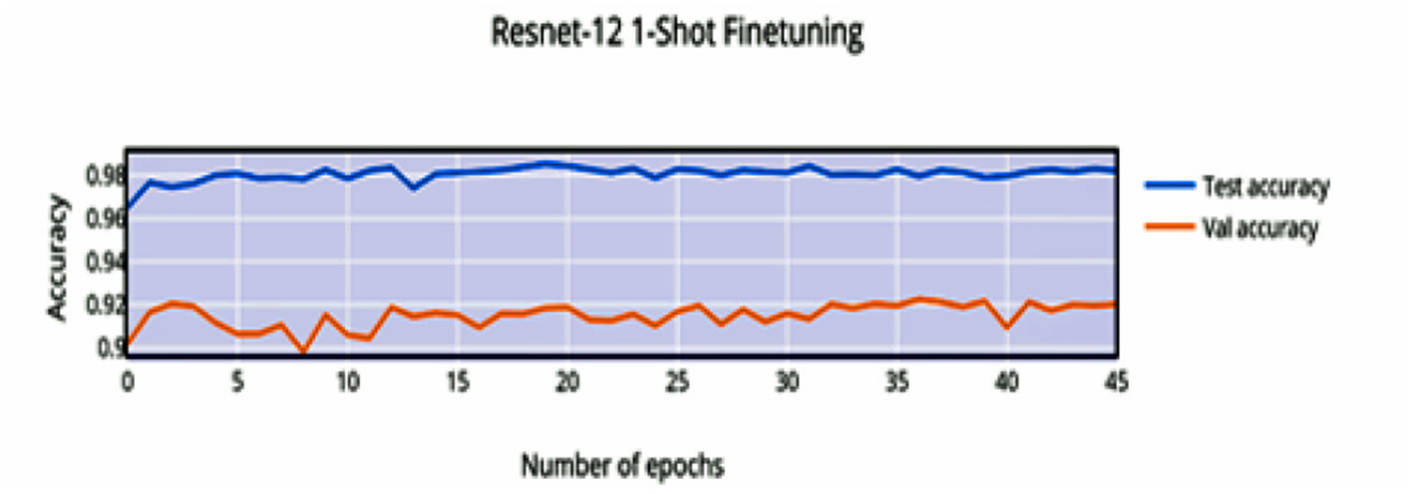
1-shot finetuning accuracy vs. number of epochs.

**Figure 9:**
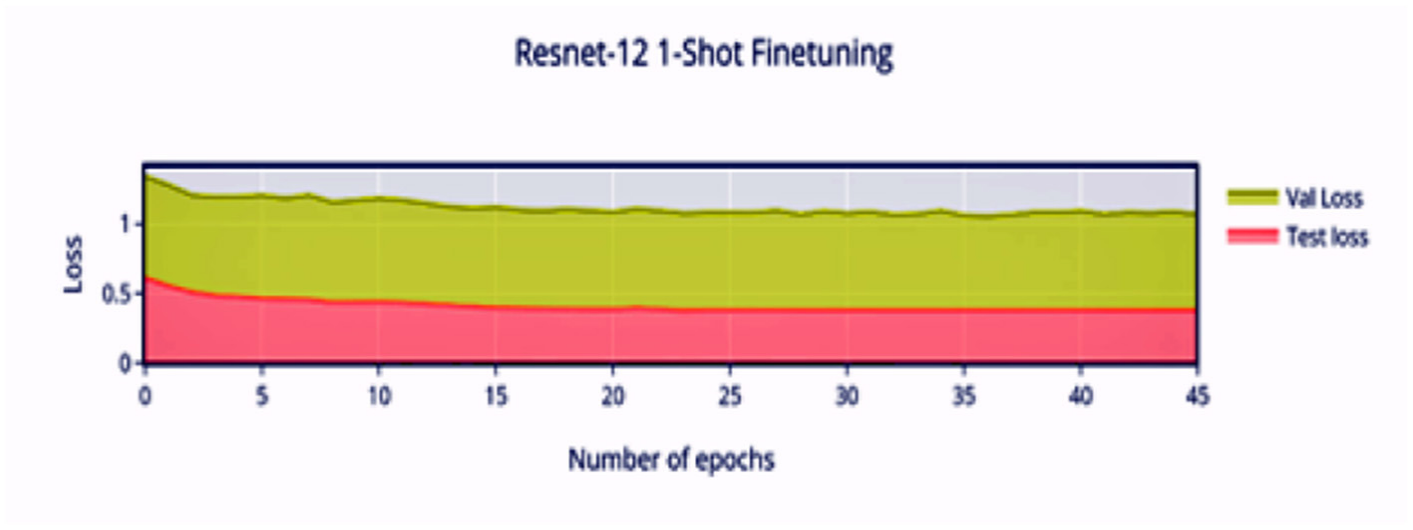
1-shot finetuning loss vs. number of epochs.

**Figure 10:**
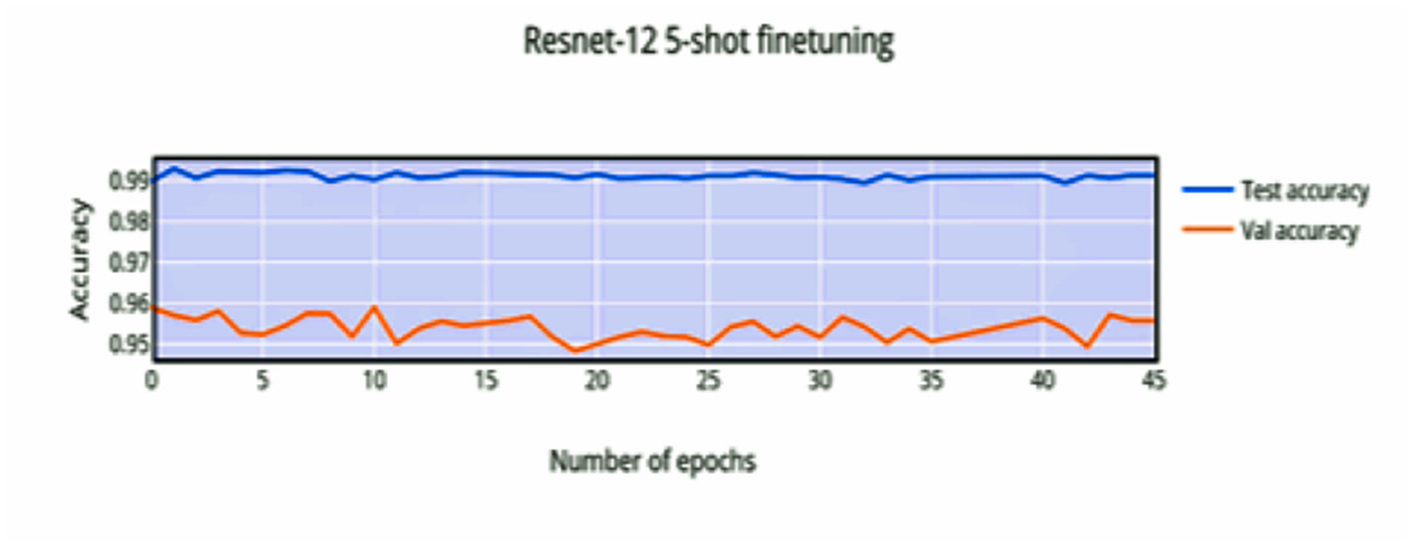
5-shot finetuning accuracy vs. number of epochs.

**Figure 11:**
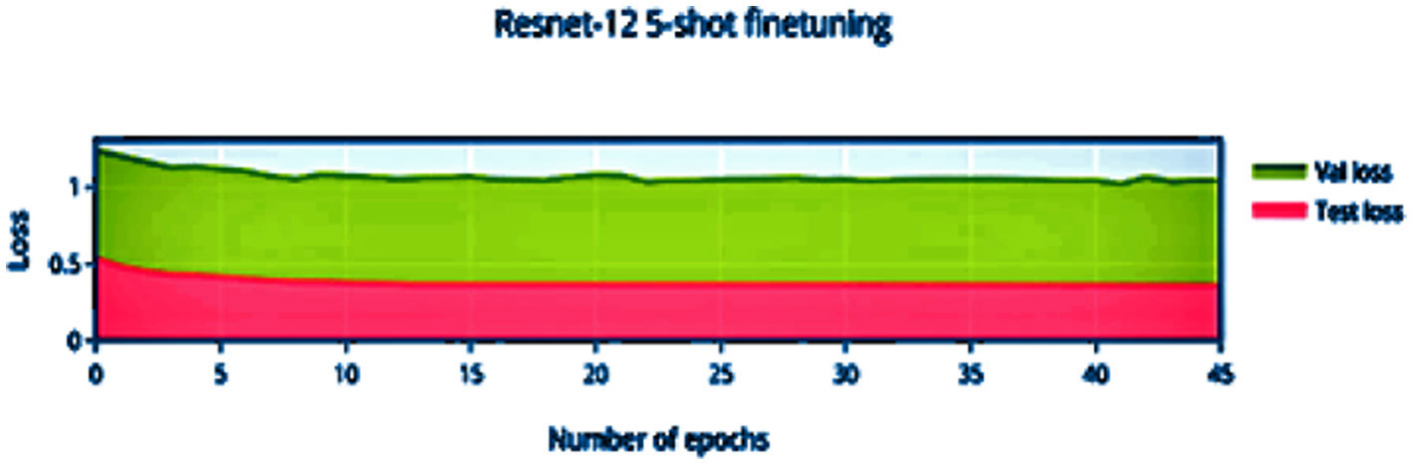
5-shot finetuning loss vs. number of epochs.

Compared to Figure 8, the finetuning does not increase the accuracy of the network by a significant amount. This can be attributed to the stronger convergence during training, which allows better inference on novel classes without much finetuning required. The low variance of the accuracy and loss in Figures 9, 11 indicates saturation of the network. Similar to Figures 10, 11, it can be observed that finetuning doesn't increase the accuracy significantly. Due to the large number of support images (5 compared to 1 in 1-shot), we obtain a higher accuracy 99.13% compared to 98.17% in Figure 11.

### 6.4 Comparison between the networks

**Figure 12:**
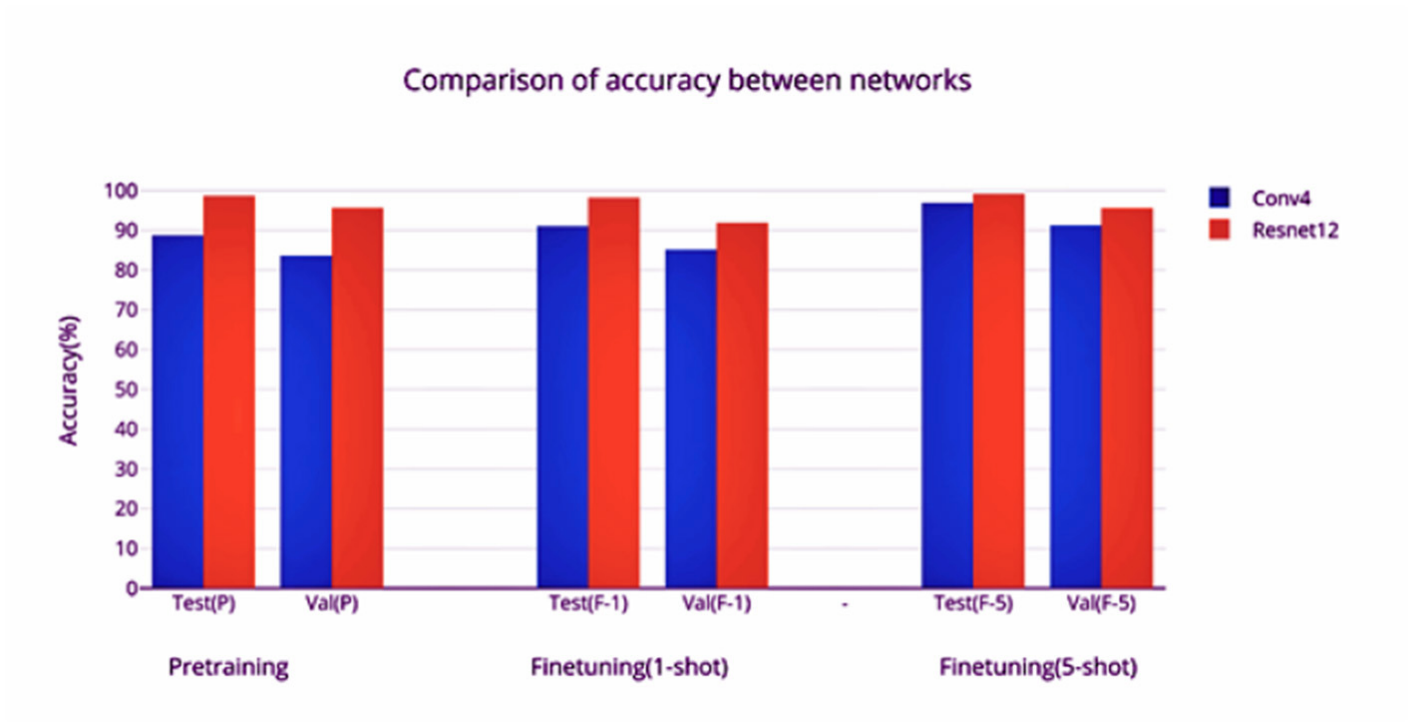
Comparison of the accuracies obtained by the networks.

The Resnet model converges faster in pretraining compared to the Conv4 model. The training is stopped when the learning rate reaches 0.00001. The learning rate is reduced to 10% after every 10 epochs if there is no improvement in the loss (a plateau is reached). A Conv4 model requires a larger number of epochs to converge during the finetuning phase as well-compared to the Resnet model (Figures 4, 8). It can be observed that there is a significant increase of test and validation accuracy during finetuning for the Conv4 model (Figures 4, 10), while finetuning doesn't increase the accuracy of the Resnet-12 model by a significant amount (Figures 9, 11). The increase in the number of support set samples from 1 to 5 provides a boost of 5% accuracy for the Conv4 model and 4% for the Resnet-12 model (comparing the validation accuracies). It can be inferred that increasing the number of labeled examples for the unseen classes can be expected to provide about a 4% increase in accuracy. The gain per increase of labeled examples should diminish as it converges to supervised learning. The comparison between the networks based on the different accuracies obtained is shown in Figure 12.

### 6.5 Comparison with previous works

The test accuracy and validation accuracy of the 5-shot approach are compared with the values obtained by training the Convolutional Neural Network and Capsule Network as provided in Ramesh et al. (2019a), as mentioned. It can be observed that the number of epochs required for convergence is similar for all three networks. The amount of overfitting in the Label Propagation network is lower as indicated by the 3% difference between the training and validation accuracies, as mentioned in Table 3. Compared to the 7% difference in the capsule network and 12% difference in the CNN used in Vinotheni and Lakshmana Pandian (2023).

**Table 3:** Comparison of accuracy with existing work.

**References**	**Method**	**Accuracy obtained**
Karthik and Srikanta Murthy (2018)	Deep belief network	97.04%
Rasheed et al. (2022)	AlexFT	97.08%
Vinotheni and Lakshmana Pandian (2023)	ETEDL-THDR	98.48%
Proposed method (5 shot)	Manifold smoothing with label propagation	99.13%

## 7 Conclusion

A novel offline handwritten character recognition framework is proposed that has the qualities of robustness to variations in input and easy generalization. The incorporation of unseen character classes into the framework doesn't require the retraining of the entire network to achieve good accuracy. The incorporation is also data efficient as it only requires a small number of labeled samples to learn to classify the newer classes (only 1 example in 1-shot and five examples in 5-shot). The use of Resnet-12 (a deep residual CNN), label propagation, and manifold smoothing helps reduce the effect of training class imbalance bias as well as reduce the overfitting of the network during the pretraining phase. The accuracy as obtained at 99.13% on the 5-shot accuracy makes this framework competitive with its supervised learning counterparts, despite the large reduction in the number of labeled samples available (for the novel classes). The framework can be further enhanced by improving the matrix inversion complexity by introducing block-sparse and sparse inversion techniques, which allow for scalability. The incorporation of the label propagation algorithm into an LSTM and language model system will help in creating few-shot learning-based word, sentence, and document optical character recognition systems.

## Data availability statement

The raw data supporting the conclusions of this article will be made available by the authors, without undue reservation.

## Author contributions

JS: Supervision, Writing - review & editing. GR: Conceptualization, Formal analysis, Investigation, Methodology, Writing - original draft. JB: Data curation, Formal analysis, Investigation, Writing - original draft. GS: Investigation, Software, Writing - original draft. HG: Project administration, Resources, Supervision, Validation, Writing - review & editing. NS: Formal analysis, Methodology, Writing - review & editing. SA: Conceptualization, Funding acquisition, Investigation, Writing - review & editing. MA: Funding acquisition, Visualization, Writing - review & editing.

## Supplementary material

Supplementary Figure 1 Diagram of the conv-4 Model.

Supplementary Figure 2 Diagram of the resent-12 Model.

Supplementary Figure 3 Gaussian similarity function.

Supplementary Figure 4 Graph clustering from power iteration.

Supplementary Figure 5 Pretraining accuracy vs. number of epochs resnet 12.

Supplementary Figure 6 Pretraining accuracy vs. number of epochs.

Supplementary Figure 7 Pretraining loss vs. number of epochs resnet 12.

Supplementary Figure 8 Pretraining loss vs. number of epochs.

Supplementary Figure 9 Samples from C train.

Supplementary Figure 10 Samples from ctest.

Supplementary Figure 11 Samples from cval.
