# Supplementary figures and images for: Hybrid manifold smoothing and label propagation technique for Kannada handwritten character recognition
Source: Front Neurosci. 2024 Apr 12;18:1362567. doi: 10.3389/fnins.2024.1362567 (PMC11045937; doi:10.3389/fnins.2024.1362567)

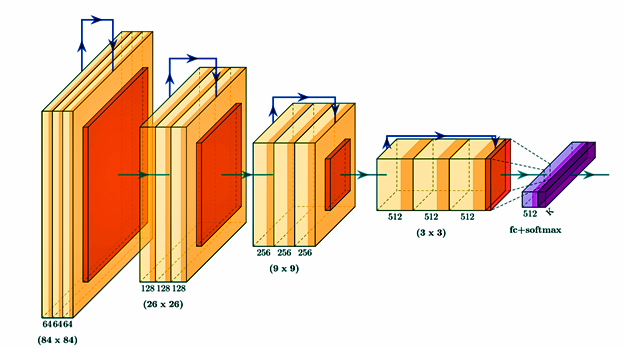

Supplement: Supplementary Figure 1 — Diagram of the conv-4 Model. [file Image_1.png]

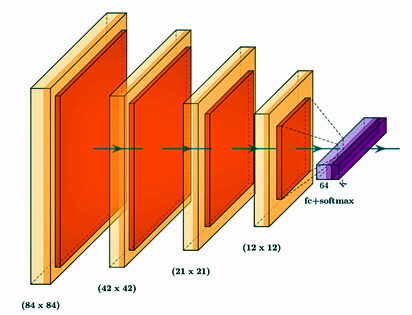

Supplement: Supplementary Figure 2 — Diagram of the resent-12 Model. [file Image_2.png]

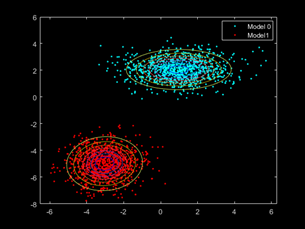

Supplement: Supplementary Figure 3 — Gaussian similarity function. [file Image_3.png]

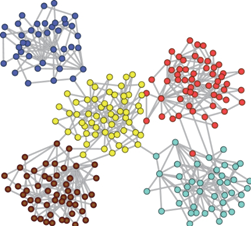

Supplement: Supplementary Figure 4 — Graph clustering from power iteration. [file Image_4.png]

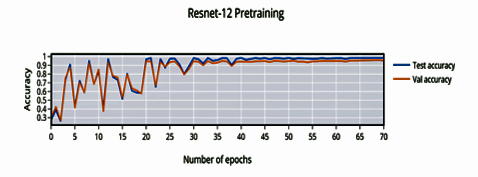

Supplement: Supplementary Figure 5 — Pretraining accuracy vs. number of epochs resnet 12. [file Image_5.png]

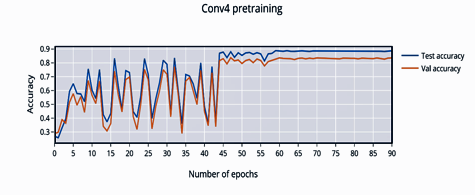

Supplement: Supplementary Figure 6 — Pretraining accuracy vs. number of epochs. [file Image_6.png]

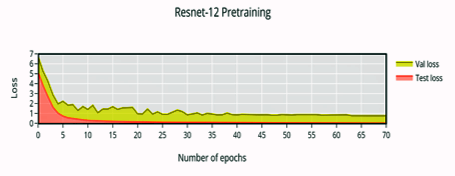

Supplement: Supplementary Figure 7 — Pretraining loss vs. number of epochs resnet 12. [file Image_7.png]

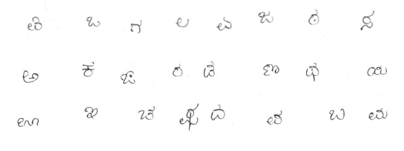

Supplement: Supplementary Figure 9 — Samples from C train. [file Image_9.png]

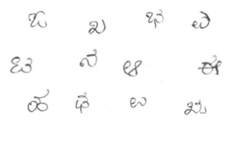

Supplement: Supplementary Figure 10 — Samples from ctest. [file Image_10.png]

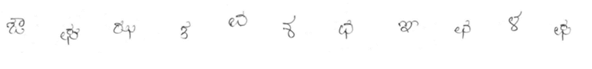

Supplement: Supplementary Figure 11 — Samples from cval. [file Image_11.png]
